# Prediction of malignant glioma grades using contrast-enhanced T1-weighted and T2-weighted magnetic resonance images based on a radiomic analysis

**DOI:** 10.1038/s41598-019-55922-0

**Published:** 2019-12-19

**Authors:** Takahiro Nakamoto, Wataru Takahashi, Akihiro Haga, Satoshi Takahashi, Shigeru Kiryu, Kanabu Nawa, Takeshi Ohta, Sho Ozaki, Yuki Nozawa, Shota Tanaka, Akitake Mukasa, Keiichi Nakagawa

**Affiliations:** 1 Department of Radiology, The University of Tokyo Hospital, 7-3-1 Hongo, Bunkyo-ku, Tokyo 113-8655 Japan; 2 Research Fellow of Japan Society for the Promotion of Science, 5-3-1 Kojimachi, Chiyoda-ku, Tokyo 102-0083 Japan; 3 Department of Medical Image Informatics, Tokushima University, 3-18-15 Kuramoto-cho, Tokushima, 770-8503 Japan; 4 Department of Neurosurgery, The University of Tokyo Hospital, 7-3-1 Hongo, Bunkyo-ku, Tokyo 113-8655 Japan; 5 Department of Radiology, International University of Health and Welfare Hospital, 537-3 Iguchi, Nasushiobara, Tochigi 329-2763 Japan; 6 Department of Neurosurgery, Graduate School of Medical Sciences, Kumamoto University, 1-1-1 Honjo, Chuo-ku, Kumamoto 860-8556 Japan

**Keywords:** Diagnostic markers, CNS cancer

## Abstract

We conducted a feasibility study to predict malignant glioma grades via radiomic analysis using contrast-enhanced T1-weighted magnetic resonance images (CE-T1WIs) and T2-weighted magnetic resonance images (T2WIs). We proposed a framework and applied it to CE-T1WIs and T2WIs (with tumor region data) acquired preoperatively from 157 patients with malignant glioma (grade III: 55, grade IV: 102) as the primary dataset and 67 patients with malignant glioma (grade III: 22, grade IV: 45) as the validation dataset. Radiomic features such as size/shape, intensity, histogram, and texture features were extracted from the tumor regions on the CE-T1WIs and T2WIs. The Wilcoxon–Mann–Whitney (WMW) test and least absolute shrinkage and selection operator logistic regression (LASSO-LR) were employed to select the radiomic features. Various machine learning (ML) algorithms were used to construct prediction models for the malignant glioma grades using the selected radiomic features. Leave-one-out cross-validation (LOOCV) was implemented to evaluate the performance of the prediction models in the primary dataset. The selected radiomic features for all folds in the LOOCV of the primary dataset were used to perform an independent validation. As evaluation indices, accuracies, sensitivities, specificities, and values for the area under receiver operating characteristic curve (or simply the area under the curve (AUC)) for all prediction models were calculated. The mean AUC value for all prediction models constructed by the ML algorithms in the LOOCV of the primary dataset was 0.902 ± 0.024 (95% CI (confidence interval), 0.873–0.932). In the independent validation, the mean AUC value for all prediction models was 0.747 ± 0.034 (95% CI, 0.705–0.790). The results of this study suggest that the malignant glioma grades could be sufficiently and easily predicted by preparing the CE-T1WIs, T2WIs, and tumor delineations for each patient. Our proposed framework may be an effective tool for preoperatively grading malignant gliomas.

## Introduction

Gliomas are primary brain tumors caused by glial cell mutations. The latest reports from the brain tumor registry of Japan indicate that 27% of brain tumor patients in Japan suffered from gliomas between 2005–2008^1^. Gliomas are classified into four grades in accordance with the pathology and genotypic figures issued by the World Health Organization (WHO)^2^. A surgical approach of removing the visible tumor tissue is typically applied to all glioma grades after imaging diagnosis based on computed tomography (CT), magnetic resonance (MR), and positron emission tomography (PET) images. Adjuvant therapy (namely chemotherapy, radiotherapy, or chemoradiotherapy) after surgery is used to treat high-grade gliomas (HGGs) to address the inevitable extension of tumors beyond margins suggested by imaging^3^. The glioma grade is determined based on pathological and genetic features of the tissues. Although an imaging diagnosis is preoperatively performed to approximate the malignancy of the tumor, the grade is usually determined based on the tissue obtained from a biopsy or resection during surgery. Glioma grading using medical imaging should be performed prior to surgery for increasing treatment effects while decreasing adverse events. In addition, predicting glioma grades using preoperative images is useful for patient education before surgery.

Methodologies for predicting glioma grades using MR or CT images have been described in previous studies^4–11^. One concept for predicting the glioma grade is to construct statistical models using some tumor appearance features or imaging indices. A more comprehensive analysis using more quantitative imaging features may provide better accuracy in predicting glioma grades. For this reason, we investigated the feasibility of radiomics in predicting glioma grades.

Radiomics is a comprehensive analysis for describing tumor phenotypes based on high-dimensional quantitative features extracted from the large quantity of medical images collected^12–14^. It has the potential to be an effective tool for personalized medicine based on phenotypic descriptions of tumors from medical images^12^, allowing for noninvasive analysis of tumor characteristics comparable with molecular biological approaches such as genomics, epigenomics, transcriptomics, and proteomics^12^. Some studies for predicting glioma grades based on radiomics using MR images have been conducted^15–22^. Qin *et al*., Cho *et al*., Chen *et al*., and Vamvakas *et al*. proposed frameworks for classifying low-grade gliomas (LGGs) and HGGs using images acquired by multiple MR imaging (MRI) sequences^15–19^. Predicting LGGs and HGGs could be made possible by constructing radiomics-based classifiers using machine learning (ML) algorithms in those frameworks. Zacharaki *et al*. and Tian *et al*. investigated the prediction of grade III and IV gliomas as well as the classification of LGGs and HGGs using images acquired via multiple MRI sequences^20,21^. Zhang *et al*. investigated both the classification of LGGs and HGGs and the prediction of grade II, III, and IV gliomas^22^. However, in previous studies, all of which used multiple MRI sequences, tumors needed to be contoured on each MR image for radiomic analysis of each patient, indicating that radiomic analysis for grading gliomas could not be performed unless all images acquired by the multiple MRI sequences were prepared in this manner. Considerable time and effort would be required to prepare tumor contours on multiple MRI sequences images for all the patients in the database. In addition, if the images acquired by a special MRI sequence were used for a framework for glioma grading based on radiomics, the framework would not have versatility for use in other institutions. Therefore, predicting the glioma grade before surgery in a straightforward manner using a few structural MRI sequences images usually acquired by the majority of institutions and volumes of interest of the tumor regions in each patient is crucial. Reza *et al*. verified the effect of three structural MRI sequences images (contrast-enhanced T1-weighted MR images (CE-T1WIs), T2-weighted MR images (T2WIs), and fluid attenuated inversion recovery (FLAIR) images) for classifying the LGGs and HGGs, and LGGs and grade IV gliomas using a few datasets^23^. However, there would be no radiomic study for verifying the effect of a few structural MRI sequences images for predicting malignant glioma grades (namely grades III and IV) using various ML algorithms.

Therefore, the purpose of this study was to investigate the feasibility of predicting malignant glioma grades based on radiomic analysis using the CE-T1WIs and T2WIs acquired before surgery.

## Materials and Methods

### Overall study design

**Figure 1:**
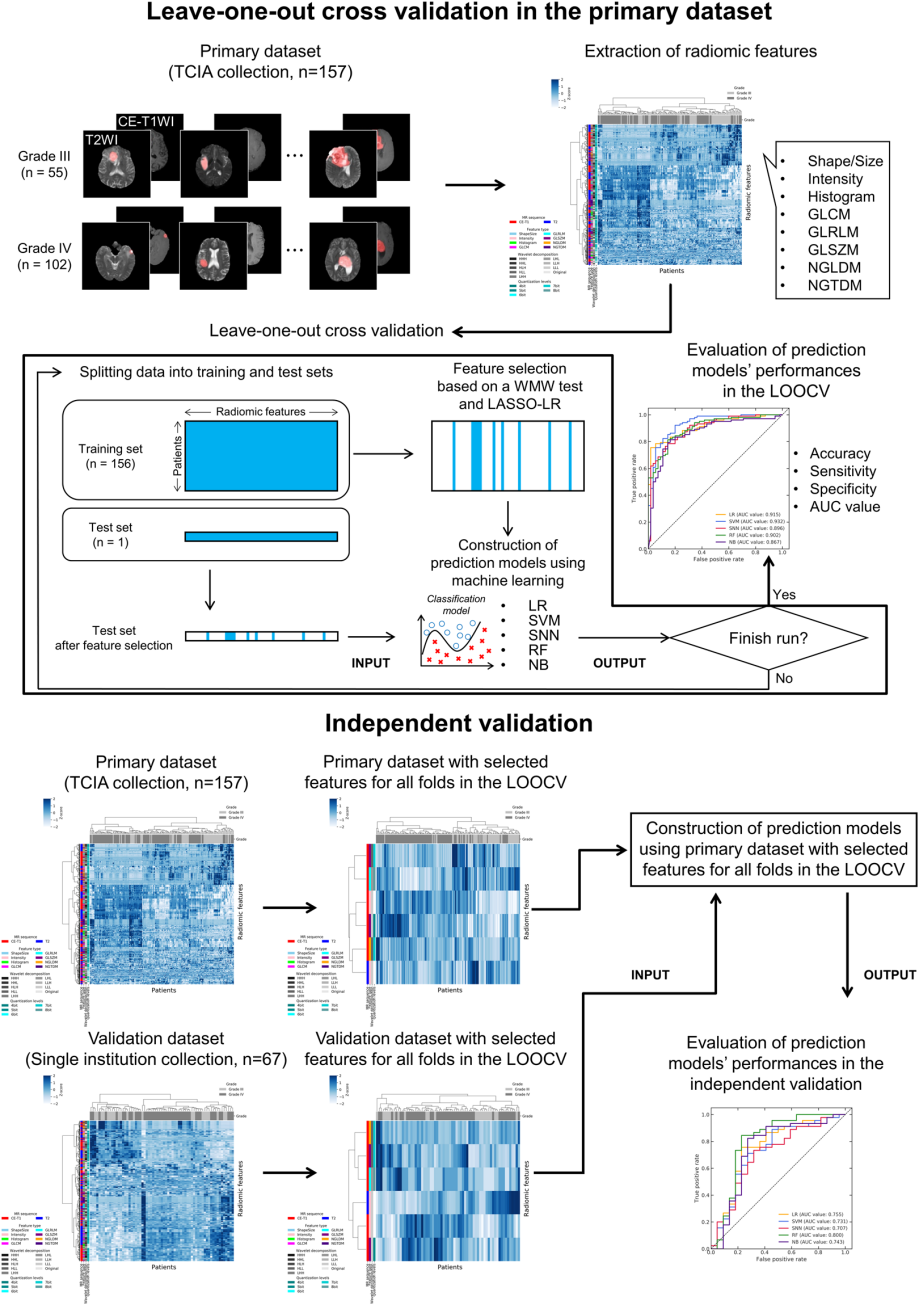
A conceptual design for predicting glioma grades based on radiomic features.

Figure 1 shows a conceptual design for predicting glioma grades based on radiomic features. The database in this study consisted of primary dataset collected in public database and validation dataset collected in our hospital. The high-dimensional radiomic features were extracted from tumor regions on the CE-T1WIs and T2WIs for all patients in the primary and validation datasets. A Wilcoxon–Mann–Whitney (WMW) test and least absolute shrinkage and selection operator logistic regression (LASSO-LR) were employed for selecting the extracted radiomic features to construct prediction models using features potentially related to glioma grades. The prediction models were constructed using the LR, a support vector machine (SVM), a standard neural network (SNN), a random forest (RF), and a naïve Bayes (NB). A leave-one-out cross-validation (LOOCV) was undertaken for evaluating the performance of the prediction models in the primary dataset. Finally, an independent validation was performed using the primary and validation datasets with selected radiomic features for all folds in the LOOCV of the primary dataset.

### Database and equipment

This study was performed in accordance with relevant guidelines and regulations approved by the institutional review board at the University of Tokyo hospital. Ethical approval for the study was also provided by the review board (reference number: 11770-[1]). Written informed consent was obtained from all subjects within the validation dataset collected in our hospital.

The brain CE-T1WIs and T2WIs archived in the cancer genome atlas glioblastoma multiforme (TCGA-GBM)^24^ and low-grade glioma (TCGA-LGG)^25^ collections of the cancer imaging archive (TCIA)^26^ were used in this study. Specifically, 157 malignant glioma patients’ preoperative CE-T1WIs and T2WIs (grade III: 55, grade IV: 102) with tumor segmentations, which were distributed via a third-party analysis using TCGA-GBM and TCGA-LGG collections^27–29^, were used as the primary dataset. The CE-T1WIs and T2WIs distributed by the third-party analysis using these collections have been transformed into the same coordinate system and interpolated to 1-mm^3^ isotropic voxels^29^. The tumor segmentations were delineated using a computerized framework and corrected by a neuroradiologist^29^. In the segmentations, there were three types of labels: (i) non-enhanced tumor and necrosis, (ii) enhanced tumor, and (iii) edema region^29^. Cho *et al*. verified that in accordance with their results, the enhanced and non-enhanced regions should be taken into account for grading the LGGs and HGGs^17^. Therefore, the tumor segmentations excluding the edema regions were used in this study. TCGA-LGG and TCGA-GBM are multicentered collections. Then, the imaging information and patients’ characteristic have been mentioned in the cited articles^24,25,27–29^.

The validation dataset comprised brain CE-T1WIs and T2WIs (with tumor region data) acquired preoperatively from 67 malignant glioma patients in our hospital. The mean number of days between image acquisition and surgery for all patients was 13.7 (range: 1–67). None of the patients underwent any treatment prior to the image acquisition that could influence the intensity of the MR images. Table 1 lists the patients’ characteristics in the validation dataset for this study. There were 22 grade III (anaplastic astrocytoma (AA): 8, anaplastic oligodendroglioma (AO): 9, anaplastic oligoastrocytoma (AOA): 5) and 45 glioblastoma (GBM) grade IV patients. The isocitrate dehydrogenase (IDH) mutation and O^6^-methylguanine-DNA methyltransferase (MGMT) methylation statuses for the GBM patients are listed in Table 1. The CE-T1WIs and T2WIs were acquired using 3.0-T MR scanners (Signa^®^ HDx and HDxt, GE Healthcare, Chicago, IL, USA). The CE-T1WIs were acquired after bolus injection of gadolinium-based contrast agents. The ranges of the repetition time (TR)/echo time (TE) for all CE-T1WIs were 380–640 ms/8–12 ms. The matrix size, pixel size, slice thickness, and spacing between the slices of the CE-T1WIs were 256 × 256, 0.82 × 0.82 mm^2^, 5.0 mm, and 6.0 mm, respectively. In the T2WIs, the range of TR/TE, matrix size, pixel size, slice thickness, and spacing between slices were 4320–4640 ms/80.77–89.28 ms, 512 × 512, 0.41 × 0.41 mm^2^, 3.0 mm, and 3.0 mm, respectively. The bit depth of the MR images was 16 bits per pixel (bpp). The CE-T1WIs and T2WIs were transformed into the same coordinate system using ITK-SNAP (ver. 3.6). A radiation technologist (T.N.) manually delineated the tumors excluding the edema regions on the MR images for all patients to extract the radiomic features; this delineation was performed under the supervision of a radiation oncologist (W.T.) and a radiologist (S.K.) for quality assurance. A commercial radiation treatment planning system (Monaco^®^ ver. 5.11, Elekta, Stockholm, Sweden) was used for the tumor delineations.

**Table 1:** Patients’ characteristics in the validation dataset for this study.

Characteristic	Value
Total number of patients	67
Gender	Male: 45 (67.2%)
	Female: 22 (32.8%)
Mean age	55.2 ± 16.2 (range: 11–83)
Grade	III: 22 (32.8%)
	IV: 45 (67.2%)
Histological type	GBM: 45 (67.2%)
	AA: 8 (11.9%)
	AO: 9 (13.4%)
	AOA: 5 (7.5%)
IDH mutation status in GBM (n = 45)	Mutated: 2 (4.4%)
	Wild type: 19 (42.2%)
	Unknown: 24 (53.3%)
MGMT methylation status in GBM (n = 45)	Methylated: 7 (15.6%)
	Unmethylated: 13 (28.9%)
	Unknown: 25 (55.6%)

GBM: glioblastoma, AA: anaplastic astrocytoma, AO: anaplastic oligodendroglioma, AOA: anaplastic oligoastrocytoma, IDH: isocitrate dehydrogenase, MGMT: O^6^-methylguanine-DNA methyltransferase.

The radiomic analysis was performed using a commercial numerical programming language (MATLAB^®^ ver. R2017a and R2017b, MathWorks, Natick, MA, USA) and an open-source numerical programming language (Python^®^ ver. 3.6). There were accessed on two workstations, one with a single 2.26 GHz quad-core central processing unit (CPU) (Intel^®^ Xeon^®^ E5607, Intel Corp., Santa Clara, CA, USA) and the other with double 2.67 GHz quad-core CPUs (Intel^®^ Xeon^®^ X5550). Both workstations had 16 GB of RAM.

### Radiomic features

**Figure 2:**
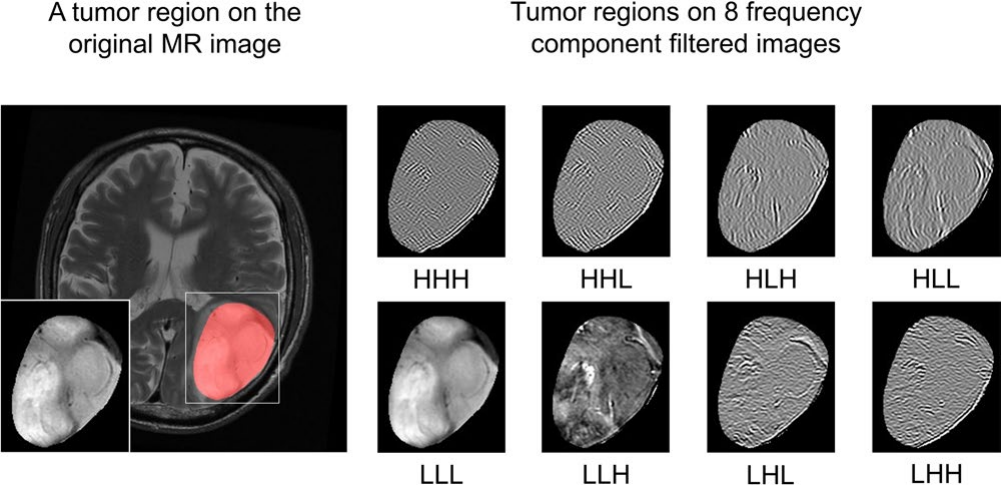
Transverse images of a tumor on original magnetic resonance (MR) image (T2-weighted MR image (T2WI)) and on eight frequency component-filtered images to which a three-dimensional (3D) Coiflet wavelet transform had been applied.

**Figure 3:**
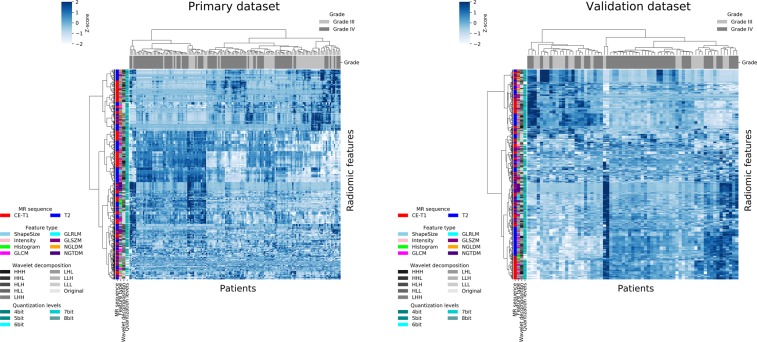
Heat maps of radiomic features in primary and validation datasets.

The radiomic features were extracted from the glioma regions on the CE-T1WIs and T2WIs using open-source MATLAB code developed by Vallières *et al*.^30,31^ (https://github.com/mvallieres/radiomics and https://github.com/mvallieres/radiomics-develop). Intensity normalization was performed for whole brain regions of the MR images in the primary and validation datasets using Z-score transformation^32^. The voxels of the MR images in the validation dataset were converted to 1-mm^3^ isotropic voxels using cubic interpolation before extracting the radiomic features. The interpolation for binary images proposed by Herman *et al*.^33^ was employed to isotropically resample the voxels of tumor mask images derived from the tumor delineation data in the validation dataset. The quantitative image features described in the image biomarker standardization initiative (IBSI)^34^ were used in this radiomic analysis. In this study, 8 shape/size features, 18 intensity features, 20 histogram features, 11 gray-level co-occurrence matrix (GLCM) features, 13 gray-level run length matrix (GLRLM) features, 13 gray-level size zone matrix (GLSZM) features, 16 neighboring gray-level dependence matrix (NGLDM), and 5 neighborhood gray-tone difference matrix (NGTDM) features within the IBSI, which have been widely used in radiomic analyses, were adopted as the radiomic features. The details of the radiomic features are provided in Supplement 1. A three-dimensional (3D) Coiflet wavelet transform^35^ was applied to the MR images in order to extract the intensity features, histogram features, and GLCM, GLRLM, GLSZM, NGLDM, and NGTDM features known as texture features in frequency decomposed images. The frequency components were HHH, HHL, HLH, HLL, LHH, LHL, LLH, and LLL, where “H” and “L” denote high-pass and low-pass filters, respectively. Thus, the intensity, histogram, and texture features were extracted from the tumor region on the original MR images and eight frequency component-filtered images. Figure 2 shows transverse images of a tumor on the original MR image (T2WI) and on eight frequency component-filtered images to which the 3D Coiflet wavelet transform had been applied. The number of bins for the histogram features was set to 6 bit. The tumor regions on the original MR images and filtered images were quantized to calculate the texture features. The quantization was performed range of μ ± 3σ, where μ and σ denote the mean and standard deviation (SD) of the voxel values in the tumor regions, respectively^36^. The quantization levels were set to 4, 5, 6, 7, and 8 bit. Figure 3 shows the heat maps of the radiomic features in the primary and validation datasets. The total number of radiomic features was 5912. The radiomic features were normalized by Z-score transformation and clustered using Ward’s method^37^ in these heat maps.

### Feature selection

**Figure 4:**
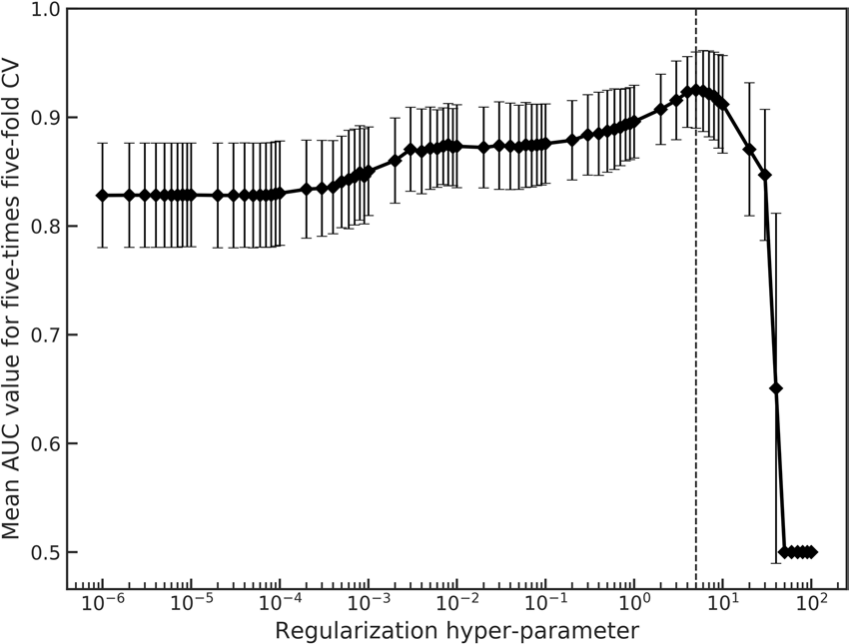
Mean area under the curve (AUC) values for five-times five-fold cross-validation (CV) for each value of a regularization hyper-parameter. The dashed line depicts a hyper-parameter value, which maximizes the mean AUC value for five-times five-fold CV.

Among the extracted radiomic features, some features would not correlate with the malignant glioma grading. Overfitted models for glioma grading would be constructed owing to these uncorrelated radiomic features. Therefore, radiomic features were selected using the WMW test and LASSO-LR^38,39^ to construct robust prediction models of the glioma grades. The two-tail WMW test was performed for all extracted radiomic features to obtain significant radiomic features (*P* < 0.001) for grading gliomas. Then, the significant radiomic features were utilize to select features using the LASSO-LR. A scikit-learn (ver. 0.19), open ML library for Python^40^ was used for the LASSO-based feature selection. The LASSO-LR can construct a classification model with sparse explanatory variables by solving an L1-norm regularized objective function expressed as follows:

1 $$\hat{{\boldsymbol{\beta }}}={\rm{\arg }}\mathop{\min }\limits_{{\boldsymbol{\beta }}}\mathop{\sum }\limits_{i=1}^{n}[-{y}_{i}\,\mathrm{ln}(h({{\bf{x}}}_{i},{\boldsymbol{\beta }}))-(1-{y}_{i})\mathrm{ln}(1-h({{\bf{x}}}_{i},{\boldsymbol{\beta }}))]+\lambda {\Vert {\boldsymbol{\beta }}\Vert }_{1},$$

where

2 $$h({{\bf{x}}}_{i},{\boldsymbol{\beta }})=\frac{1}{1+\exp (-{{\boldsymbol{\beta }}}^{{\rm{T}}}{{\bf{x}}}_{i})},$$

3 $${{\bf{x}}}_{i}=({x}_{1,i},{x}_{2,i},\,\ldots \,,{x}_{p,i}),$$

4 $${\boldsymbol{\beta }}=({\beta }_{1},{\beta }_{2},\,\ldots ,\,{\beta }_{p}),$$

where $\hat{{\boldsymbol{\beta }}}$ is an optimal coefficient vector, *n* is the number of patients, *y* is a label for the glioma grades, and *λ* is a hyper-parameter of the regularization. **x**, **β**, and *p* are explanatory vectors comprising the significant radiomic features, coefficient vector, and number of the significant radiomic features, respectively. The optimization problem was solved using a coordinate descent algorithm^41^. $\hat{{\boldsymbol{\beta }}}$ would be a sparse vector owing to L1-norm regularization. The features with non-zero coefficients of the $\hat{{\boldsymbol{\beta }}}$ were selected in this study*. λ*, the hyper-parameter determining the regularization effect in the optimization problem^42^, was tuned in this study by using a grid search technique. In the grid search, five-fold cross-validation (CV) was performed five times in the training set while changing the values of the hyper-parameter, and mean values for the area under receiver operating characteristic (ROC) curve (or simply the area under the curve (AUC)) for the five-times five-fold CV were calculated for each value of the hyper-parameter. The value of the hyper-parameter that maximized the mean AUC value for the five-times five-fold CV was used for the regularization Figure 4 shows the mean AUC values for the five-times five-fold CV for each value of the regularization hyper-parameter. The range of the hyper-parameter values was 10^−6^–10^2^.

### Construction of prediction models for glioma grades using machine learning algorithms

The scikit-learn was also used in this procedure. The LR, SVM^43^, SNN^44^, RF^45^, and NB^46^ were used to construct the prediction models for the malignant glioma grades using the selected radiomic features. Some hyper-parameters of the LR, SVM, SNN, and RF were tuned by the same methodology as that used for feature selection. The ranges for tuning the hyper-parameters by using grid search are provided in Supplement 2. In the SVM, a radial basis function kernel was used to construct nonlinear models^43^. Almost all hyper-parameters of the SNN and RF were fixed default values provided by scikit-learn^40^. In the RF, number of trees was fixed to 1000. There was no parameter for tuning in the NB. The LOOCV was conducted to evaluate the performance of prediction models derived from the LR, SVM, SNN, RF, and NB in the primary dataset. Independent validation was also performed to investigate the versatility of the radiomic analysis with a few structural MRI sequences for predicting the malignant glioma grades using the primary and validation datasets. Specifically, the prediction models were constructed using the primary dataset with the selected radiomic features for all folds in the LOOCV; then, the prediction models were evaluated using the validation dataset with the selected radiomic features. Accuracies, sensitivities, specificities, and AUC values for all prediction models were calculated as evaluation indices. Grade III and IV gliomas were defined as negative and positive, respectively, for calculating the evaluation indices.

## Results

The range and mode of the number of the significant radiomic features for grading malignant gliomas for the LOOCV were 593–717 and 638, respectively. The range and mode of the number of selected radiomic features for the LOOCV were 21–39 and 30, respectively. The mean percentage of number of selected radiomic features for the LOOCV was 0.53%. The mean ± SD of the value of the hyper-parameter of regularization for the LOOCV was 5.02 ± 0.76 (95% confidence interval (CI), 4.90–5.14). Table 2 lists the selected radiomic features for all folds in the LOOCV of the primary dataset. The number of selected radiomic features for all LOOCV folds in the CE-T1WIs and T2WIs were 5 (intensity: 1, GLRLM: 1, GLSZM: 2, NGLDM: 1), and 1 (intensity: 1), respectively.

**Table 2:** Selected radiomic features for all folds in a leave-one-out cross-validation (LOOCV) of the primary dataset.

MRI sequence	Wavelet	Quantization levels	Feature type	Feature name
CE-T1	LLL	—	Intensity	Median
CE-T1	LHL	8 bit	GLRLM	Run-length variance
CE-T1	LLL	5 bit	GLSZM	Gray-level non-uniformity normalized
CE-T1	HLL	7 bit	GLSZM	Gray-level variance
CE-T1	HLL	7 bit	NGLDM	High dependence low gray-level emphasis
T2	LLL	—	Intensity	Root mean square

CE-T1: contrast-enhanced T1, L: low-pass filter, H: high-pass filter, GLRLM: gray-level run length matrix, GLSZM: gray-level size zone matrix, NGLDM: neighboring gray-level dependence matrix.

**Figure 5:**
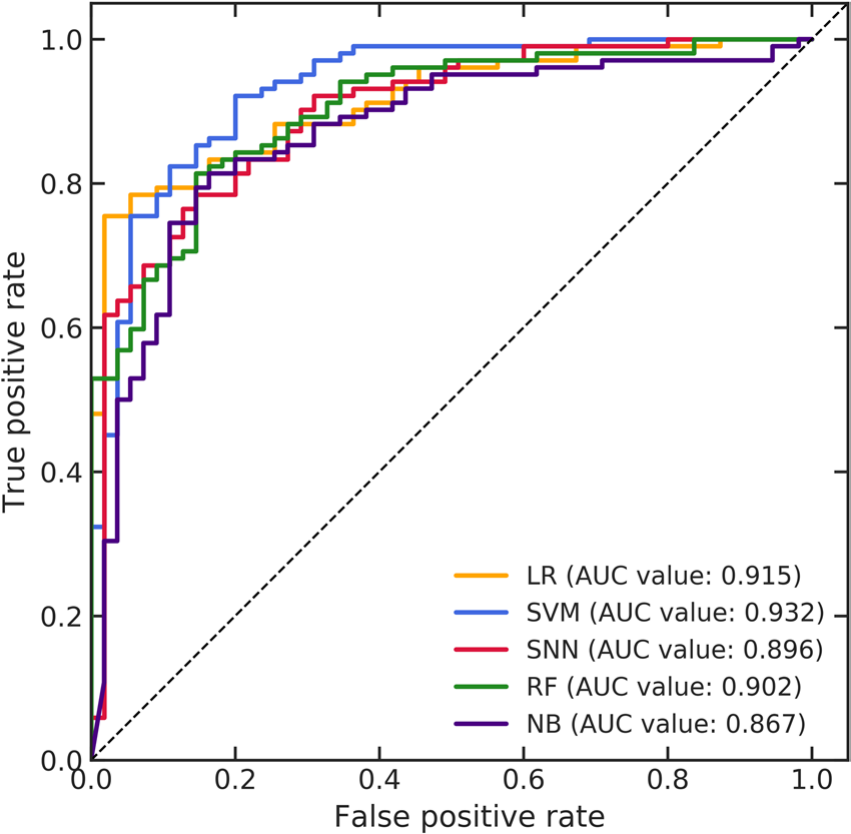
Receiver operating characteristic (ROC) curves of the prediction models constructed by the five machine learning (ML) algorithms in a leave-one-out cross-validation (LOOCV) of the primary dataset.

Figure 5 shows the ROC curves of the prediction models constructed by the five ML algorithms in the LOOCV of the primary dataset. The AUC values of the prediction models constructed by the LR, SVM, SNN, RF, and NB were 0.915, 0.932, 0.896, 0.902, and 0.867, respectively. Table 3 lists the accuracies, sensitivities, specificities, and AUC values of the prediction models in the LOOCV of the primary dataset. The mean ± SD of these four parameters for all prediction models were 0.824 ± 0.027 (95% CI, 0.790–0.858), 0.863 ± 0.033 (95% CI, 0.822–0.903), 0.753 ± 0.065 (95% CI, 0.672–0.833), and 0.902 ± 0.024 (95% CI, 0.873–0.932), respectively. The prediction models using the SVM demonstrated the best performance for classifying the malignant glioma grades in the LOOCV of the primary dataset, based on the resulting AUC value (0.932).

**Table 3:** Accuracies, sensitivities, specificities, and area under the curve (AUC) values of prediction models in a leave-one-out cross-validation (LOOCV) of the primary dataset.

Machine learning algorithm	Accuracy	Sensitivity	Specificity	AUC
LR	0.834	0.833	0.836	0.915
SVM	0.866	0.902	0.800	0.932
SNN	0.796	0.833	0.727	0.896
RF	0.815	0.892	0.673	0.902
NB	0.809	0.853	0.727	0.867
Mean ± SD	0.824 ± 0.027	0.863 ± 0.033	0.753 ± 0.065	0.902 ± 0.024
95% CI	0.790–0.858	0.822–0.903	0.672–0.833	0.873–0.932

LR: logistic regression, SVM: support vector machine, SNN: standard neural network, RF: random forest, NB: naïve Bayes.

**Figure 6:**
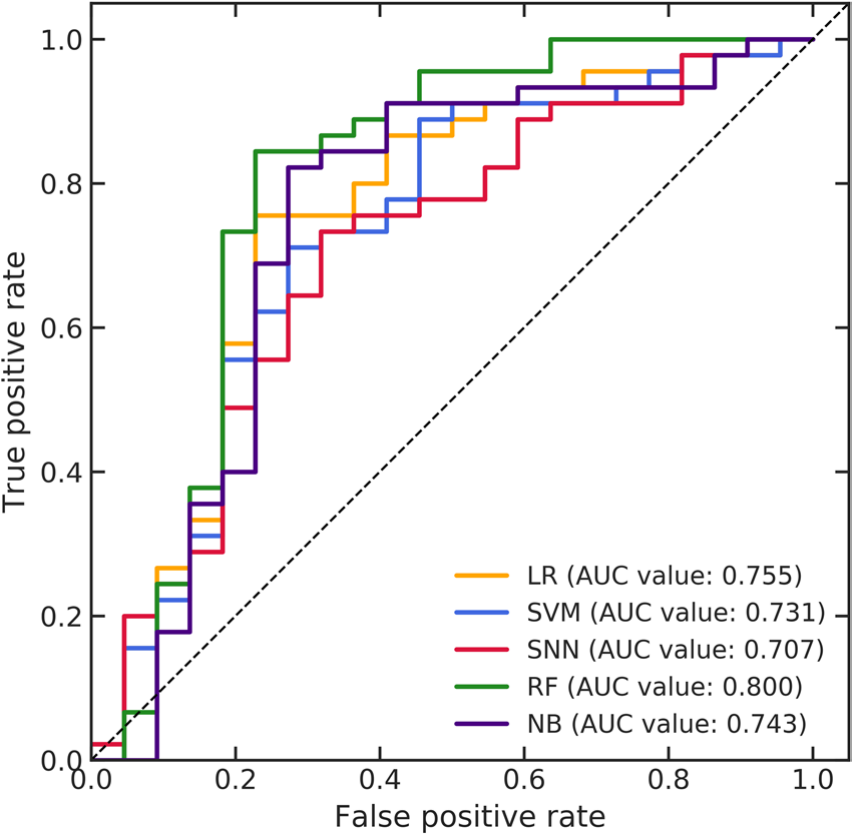
Receiver operating characteristic (ROC) curves for all prediction models in an independent validation constructed by using selected radiomic features for all folds in a leave-one-out cross-validation (LOOCV).

Figure 6 shows the ROC curves for all prediction models in the independent validation constructed by using selected radiomic features for all folds in the LOOCV. The AUC values of the prediction models constructed by the LR, SVM, SNN, RF, and NB were 0.755, 0.731, 0.707, 0.800, and 0.743, respectively. Table 4 lists the accuracies, sensitivities, specificities, and AUC values of the prediction models in the independent validation. The mean ± SD of these four parameters for all prediction models were 0.758 ± 0.034 (95% CI, 0.716–0.800), 0.822 ± 0.042 (95% CI, 0.771–0.874), 0.627 ± 0.149 (95% CI, 0.443–0.812), and 0.747 ± 0.034 (95% CI, 0.705–0.790), respectively. The prediction models using the RF demonstrated the best performance in the independent validation, based on the resulting AUC value (0.800).

**Table 4:** Accuracies, sensitivities, specificities, and area under the curve (AUC) values of prediction models in an independent validation.

Machine learning algorithm	Accuracy	Sensitivity	Specificity	AUC
LR	0.746	0.756	0.727	0.755
SVM	0.746	0.844	0.545	0.731
SNN	0.716	0.867	0.409	0.707
RF	0.806	0.822	0.773	0.800
NB	0.776	0.822	0.682	0.743
Mean ± SD	0.758 ± 0.034	0.822 ± 0.042	0.627 ± 0.149	0.747 ± 0.034
95% CI	0.716–0.800	0.771–0.874	0.443–0.812	0.705–0.790

LR: logistic regression, SVM: support vector machine, SNN: standard neural network, RF: random forest, NB: naïve Bayes.

## Discussion

The feasibility of predicting malignant glioma grades based on radiomics by using images acquired with two structural MRI sequences was investigated herein. The classification of LGGs and HGGs using MR-based radiomic frameworks has been investigated and successfully performed in the past^15–23^. However, this study is focused on only classification of the grade III and IV malignant gliomas because it is also crucial to preoperatively classify the grade IV and the others gliomas for appropriate surgical planning and prognosis prediction. The primary dataset derived from TCIA collection and the validation dataset derived from our institution collection were used to evaluate prediction performances. High-dimensional radiomic features were extracted from both CE-T1WIs and T2WIs in various feature types, wavelet sub-bands, and quantization levels to comprehensively obtain effective features for predicting the malignant glioma grades. The effective features were selected by using combination of the WMW test and LASSO-LR. Five ML algorithms were applied to construct various prediction models using the selected radiomic features for each fold in the LOOCV of the primary dataset. The primary and validation datasets with the selected radiomic features for all folds in the LOOCV of the primary dataset were utilized in the independent validation. The prediction performances of various models were compared using four evaluation indices.

The AUC values of the prediction models constructed by the LR, SVM, and RF in the LOOCV of the primary dataset reached 0.90 and those in the SNN and NB reached 0.80. Moreover, the mean AUC values for all prediction models was 0.902 ± 0.024. In general, classification models with AUC values of 1.00–0.90, and 0.90–0.80 are regarded as excellent and good, respectively^47,48^. Therefore, the proposed framework could accurately predict malignant glioma grades despite using images acquired with a few structural MRI sequences in the primary dataset. The best prediction performance in the LOOCV of the primary dataset was 0.932 of AUC value using the SVM. Therefore, the SVM was an effective classifier for predicting the grade III and IV gliomas in the primary dataset.

The radiomic features extracted from the CE-T1WIs were dominantly selected for each fold in the LOOCV. In addition, there were five radiomic features extracted from the CE-T1WIs and one radiomic feature extracted from the T2WIs, which were selected for all folds in the LOOCV using the primary dataset. The selected radiomic features for all LOOCV folds comprised almost all texture features extracted from the CE-T1WIs. Tian *et al*. reported that the texture features extracted from the CE-T1WIs contributed the most to optimal feature subsets for predicting the LGGs and HGGs and grade III and IV gliomas in the multiple MRI sequences images^21^. They then suggested that the texture features extracted from the CE-T1WIs might lead to high performance while grading the gliomas^21^. Reza *et al*. have also reported that in accordance with the results of feature importance ranking in the feature selection, the radiomic features extracted from the CE-T1WIs were more important than those extracted from other structural MRI sequences images^23^. The result of feature selection for all LOOCV folds in this study was consistent with those reports. Cho *et al*. and Vamvakas *et al*. have used 7 and 8 bit of fixed quantization levels, respectively for extracting the texture features^17,19^. Then, the values of the quantization levels have not been mentioned in almost all previous studies^15,16,18,21,22^. Few studies have been reported the appropriate values of the quantization levels for grading the gliomas. In this study, five types of values were used to have various combinations of quantization levels in the texture features for achieving high performance. The texture features derived from high quantization levels (7 and 8 bit) were dominantly selected for all folds in the LOOCV. Therefore, the texture features with the high quantization levels might be effective for predicting the malignant glioma grades.

The AUC values of the prediction models were greater than 0.70 but less than 0.80 excluding that of the model constructed by the RF in the independent validation. These results suggested that the performances for predicting the malignant glioma grades in the independent validation were acceptable but not good excluding that of the RF. In addition, the mean AUC values for all prediction models in the independent validation was lower than that in the LOOCV of the primary dataset. The prediction performance degradation in the independent validation could be attributed to the difference in observers for delineating tumors in the primary and validation datasets. The performance for the radiomic analysis varied, depending on the MR scanners, imaging parameters, and tumor delineations^49,50^. We used MR images acquired by various scanners and imaging parameters in the entire dataset. Therefore, MR intensity normalization was performed as preprocessing for the entire dataset to reduce the influences on the performances caused by those variabilities. However, in terms of delineation, the tumor regions in the primary dataset were delineated by combination of a computerized framework and manual correction by an expert^29^, while tumor regions in the validation dataset were manually delineated by an observer under the supervision of two experts. Consequently, the selected radiomic features for all folds in the LOOCV of the primary dataset could not have robustness to delineations of the difference observer. The results of independent validation suggested that reproducible radiomic features to the observer delineation variability should be investigated to obtain high prediction performance in case using difference datasets.

Previous studies^20,21^ had already proposed radiomics-based frameworks for classifying malignant glioma grades using images acquired via multiple MRI sequences. Table 5 lists the prediction performances for malignant glioma grade identification using a radiomic approach in the proposed framework and in previous studies. The best prediction performances of the LOOCV and independent validation using the CE-T1WIs and T2WIs in the proposed framework were listed in Table 5. Prediction performances with more than 0.90 of the AUC values reported by Zacharaki *et al*. were listed in Table 5 because they investigated various combinations of feature selection methods and classifiers for grading the malignant gliomas^20^. The AUC values of the previous studies with the multiple MRI sequences were higher than those of our proposed framework with a few structural MRI sequences. The frameworks of previous studies using multiple MRI sequences were indeed effective for classifying malignant glioma grades. However, there might be selection bias in the prediction performances of the previous studies owing to the relatively small datasets used compared with those of this study and using single scanner and unified parameters for acquiring MR images in the datasets. Moreover, an independent validation for investigating versatility to the different datasets was not performed in previous studies. In this study, the AUC values of the best prediction performances in the LOOCV and independent validation using datasets with variety were reached 0.90 and 0.80, respectively. Therefore, we can conclude that our proposed framework with a few structural MRI sequences could sufficiently predict malignant glioma grades despite using datasets comprising MR images acquired by various scanners and imaging parameters.

**Table 5:** Prediction performances for malignant glioma grade identification using a radiomic approach in the proposed framework and in previous studies.

Study	No. of data	MRI sequence	Feature type	Filtering	Feature selection	ML algorithm	Data augmentation	Validation method	Accuracy	Sensitivity	Specificity	AUC value
Proposed framework	Primary dataset: 157 (III: 55, IV: 102)	•CE-T1 •T2	•Shape/size •Intensity •Histogram •GLCM •GLRLM •GLSZM •NGLDM •NGTDM	Wavelet transform high-pass and low-pass filters for all feature types excluding the shape/size	WMW test & LASSO-LR	SVM (rbf kernel)	No	LOOCV	0.866	0.902	0.800	0.932
	Entire dataset: 224 (Primary dataset: 157 & Validation dataset: 67 (III: 22, IV: 45))				Using the selected radiomic features for all folds in the LOOCV of the primary dataset	RF	No	Independent validation	0.806	0.822	0.773	0.800
Zacharaki *et al*.^20^	52 (III: 18, IV: 34)	•CE-T1 •T1 •T2 •FLAIR •rCBV	•Shape •Intensity •Rotation invariant texture	Gabor filter for rotation invariant texture features	SVM-RFE	SVM (rbf kernel)	No	LOOCV	0.904	1.000	0.722	0.985
					t-test with bagging				0.942	NR	NR	1.000
Tian *et al*.^21^	111 (III: 33, IV: 78)	•CE-T1 •T1 •T2 •Diffusion •3D pCASL	•GLCM •GLGCM	No	SVM-RFE	SVM (rbf kernel)	No	100-times 10-fold CV	0.937	0.942	0.927	0.982
							SMOTE		0.981	0.987	0.974	0.992

CE-T1: contrast-enhanced T1, FLAIR: fluid attenuated inversion recovery, rCBV: relative blood volume, 3D-pCASL: three-dimensional pseudo-continuous arterial spin labeling, GLCM: gray-level co-occurrence matrix, GLRLM: gray-level run length matrix, GLSZM: gray-level size zone matrix, NGLDM: neighboring gray-level dependence matrix, NGTDM: neighborhood gray-tone difference matrix, GLGCM: gray-level gradient co-occurrence matrix, WMW: Wilcoxon-Mann-Whitney, LASSO-LR: least absolute shrinkage and selection operator logistic regression, RFE: recursive feature elimination, SMOTE: synthetic minority over sampling technique, SVM: support vector machine, rbf: radial basis function, RF: random forest, LOOCV: leave-one-out cross validation, AUC: area under the curve, NR: not reported.

There are limitations to our study. Owing to the difficulty of collecting a large number of available malignant glioma cases for a study at our institution, the number of cases in the validation dataset was small. In future, a multi-institutional study would be more helpful. Moreover, some cases lacked several MRI sequences images in the validation dataset owing to retrospective data collection. Therefore, insufficient multiple MRI sequences images were available at our institution for comparison with CE-T1WIs and T2WIs, and the prediction performances using the CE-T1WIs and T2WIs in this study were compared instead with those using multiple MRI sequence images in the previous studies. In addition, the effect of inter-observer tumor delineation variability on the prediction performances of the malignant glioma grades, the reproducible features to the delineation variability, and an appropriate tumor delineation procedure for radiomic analysis should be investigated in future. Finally, although prediction of the glioma grades using preoperative MR images would be useful for planning surgery, the genomic statuses of the gliomas (for example IDH mutation, alpha-thalassemia/mental retardation syndrome X-linked (ATRX) mutation, TP53 mutation, and 1p19q codeletion^2^) should be identified using radiomics-based analysis (namely radiogenomics) with a few structural MRI sequences for precision medicine. The genomic statuses of the gliomas were difficult to analyze in this study because genomic analyses were not always performed for all cases. In a future study, the proposed framework should be applied to prediction of the genomic features of the gliomas by collecting a large quantity of patients’ preoperative MR images and genomic statuses.

In conclusion, we investigated the feasibility of a framework for predicting malignant glioma grades based on radiomics using CE-T1WIs and T2WIs. Our proposed framework could sufficiently and easily predict malignant glioma grades by preparing images acquired by a few structural MRI sequences. The proposed framework with a few MRI sequences could mitigate the tedious process of tumor contouring on each MRI sequence image compared with the frameworks with multiple MRI sequences. In addition, the best prediction performances of this study indicated that our proposed framework with a few MRI sequences could have versatility to varied datasets. Our proposed framework for noninvasively grading malignant gliomas based on the preoperative images could be an effective tool for selection of appropriate surgery and educating the patients.

## Supplementary information

Table 1, Table 2
