# Publisher Correction: Prediction of malignant glioma grades using contrast-enhanced T1-weighted and T2-weighted magnetic resonance images based on a radiomic analysis

**DOI:** 10.1038/s41598-020-60086-3

**Published:** 2020-02-17

**Authors:** Takahiro Nakamoto, Wataru Takahashi, Akihiro Haga, Satoshi Takahashi, Shigeru Kiryu, Kanabu Nawa, Takeshi Ohta, Sho Ozaki, Yuki Nozawa, Shota Tanaka, Akitake Mukasa, Keiichi Nakagawa

**Affiliations:** 1 Department of Radiology, The University of Tokyo Hospital, 7-3-1 Hongo, Bunkyo-ku, Tokyo 113-8655 Japan; 2 Research Fellow of Japan Society for the Promotion of Science, 5-3-1 Kojimachi, Chiyoda-ku, Tokyo 102-0083 Japan; 3 Department of Medical Image Informatics, Tokushima University, 3-18-15 Kuramoto-cho, Tokushima, 770-8503 Japan; 4 Department of Neurosurgery, The University of Tokyo Hospital, 7-3-1 Hongo, Bunkyo-ku, Tokyo 113-8655 Japan; 5 Department of Radiology, International University of Health and Welfare Hospital, 537-3 Iguchi, Nasushiobara, Tochigi 329-2763 Japan; 6 Department of Neurosurgery, Graduate School of Medical Sciences, Kumamoto University, 1-1-1 Honjo, Chuo-ku, Kumamoto 860-8556 Japan

Correction to: *Scientific Reports* 10.1038/s41598-019-55922-0, published online 19 December 2019

The PDF version of this Article contained errors in Table 5. In the row entitled ‘Zacharaki *et al*.^20^’, text was omitted from the ‘MRI sequence’ column and there were errors in the ‘Feature type’ and ‘Filtering’ columns. In addition, text was also omitted from the ‘Tian *et al*.^21^’ row in the ‘MRI sequence’ column.

These errors have now been corrected in the PDF version of this Article.

