# Supplementary material for: Prediction of malignant glioma grades using contrast-enhanced T1-weighted and T2-weighted magnetic resonance images based on a radiomic analysis
Source: Sci Rep. 2019 Dec 19;9:19411. doi: 10.1038/s41598-019-55922-0 (PMC6923390; doi:10.1038/s41598-019-55922-0)
Supplement: Supplementary file 1 — Table 1, Table 2 [file 41598_2019_55922_MOESM1_ESM.docx]

**Supplementary file**

**Prediction of malignant glioma grades using contrast-enhanced T1-weighted and T2-weighted magnetic resonance images based on a radiomic analysis**

Takahiro Nakamoto^1, 2^, Wataru Takahashi^1^, Akihiro Haga^1, 3^, Satoshi Takahashi^4^, Shigeru Kiryu^5^, Kanabu Nawa^1^, Takeshi Ohta^1^, Sho Ozaki^1^, Yuki Nozawa^1^, Shota Tanaka^4^, Akitake Mukasa^6^, and Keiichi Nakagawa^1^

^1^Department of Radiology, The University of Tokyo Hospital, 7-3-1 Hongo, Bunkyo-ku, Tokyo, 113-8655, Japan

^2^Research Fellow of Japan Society for the Promotion of Science, 5-3-1 Kojimachi, Chiyoda-ku, Tokyo, 102-0083, Japan

^3^Department of Medical Image Informatics, Tokushima University, 3-18-15 Kuramoto-cho, Tokushima, 770-8503, Japan

^4^Department of Neurosurgery, The University of Tokyo Hospital, 7-3-1 Hongo, Bunkyo-ku, Tokyo, 113-8655, Japan

^5^Department of Radiology, International University of Health and Welfare Hospital, 537-3 Iguchi, Nasushiobara, Tochigi, 329-2763, Japan

^6^Department of Neurosurgery, Graduate School of Medical Sciences, Kumamoto University, 1-1-1 Honjo, Chuo-ku, Kumamoto, 860-8556, Japan

| **Supplementary Table 1** Details of radiomic features adopted in this study. | | |
| --- | --- | --- |
| Feature type | | Feature name |
| Shape/size (8) | | Compactness1, Compactness2, Max diameter, Spherical disproportion, Sphericity, Surface area, Surface volume ratio, Volume |
| Intensity (18) | | Maximum, Minimum, Mean, Median, Range, Variance, Skewness, Kurtosis, Energy, 10th percentile, 90th percentile, Interquartile range, Mean absolute deviation, Robust mean absolute deviation, Median absolute deviation, Quartile coefficient of dispersion, Root mean square, Coefficient of variation |
| Histogram (20) | | Mean, Median, Variance, Skewness, Kurtosis, Entropy, Uniformity, 10th percentile, 90th percentile, Mode, Interquartile range, Mean absolute deviation, Robust mean absolute deviation, Median absolute deviation, Quartile coefficient of dispersion, Coefficient of variation, Maximum gradient, Maximum gradient gray-level, Minimum gradient, Minimum gradient gray-level |
| Texture (58) | GLCM (11) | Energy, Contrast, Entropy, Correlation1, Correlation2, Auto correlation, Homogeneity1, Homogeneity2, Variance, Sum average, Dissimilarity |
|  | GLRLM (13) | Short run emphasis, Long run emphasis, Gray-level non-uniformity normalized, Run-length non-uniformity normalized, Run percentage, Low gray-level run emphasis, High gray-level run emphasis, Short run low gray-level emphasis, Short run high gray-level emphasis, Long run low gray-level emphasis, Long run high gray-level emphasis, Gray-level variance, Run-length variance |
|  | GLSZM (13) | Small zone emphasis, Large zone emphasis, Gray-level non-uniformity normalized, Zone-size non-uniformity normalized, Zone percentage, Low gray-level zone emphasis, High gray-level zone emphasis, Small zone low gray-level emphasis, Small zone high gray-level emphasis, Large zone low gray-level emphasis, Large zone high gray-level emphasis, Gray-level variance, Zone-size variance |
|  | NGLDM (16) | Low dependence emphasis, High dependence emphasis, Gray-level non-uniformity, Gray-level non-uniformity normalized, Dependence-count non-uniformity, Dependence-count non-uniformity normalized, Low gray-level count emphasis, High gray-level count emphasis, Low dependence low gray-level emphasis, Low dependence high gray-level emphasis, High dependence low gray-level emphasis, High dependence high gray-level emphasis, Gray-level variance, Dependence-count variance, Dependence-count entropy, Dependence-count energy |
|  | NGTDM (5) | Coarseness, Contrast, Busyness, Complexity, Strength |

GLCM: gray-level co-occurrence matrix, GLRLM: gray-level run length matrix, GLSZM: gray-level size zone matrix, NGLDM: neighboring gray-level dependence matrix, NGTDM: neighborhood gray-tone difference matrix

| **Supplementary** **Table 2** Ranges for tuning hyper-parameters by using grid search. | | |
| --- | --- | --- |
| ML algorithm | Hyper-parameter | Range of the grid search |
| LR | “C” (parameter of L2-norm regularization term) | [100, 10, 1, 0.1, 0.01, 0.001] |
| SVM (kernel: “rbf”) | “C” (parameter of L2-norm regularization term) | [100, 10, 1, 0.1, 0.01, 0.001] |
|  | “gamma” (kernel coefficient) | [100, 10, 1, 0.1, 0.01, 0.001] |
| SNN | “hidden_layer_sizes” (no. of elements: no. of hidden layers, value in the element: no. of nodes in the hidden layer) | [(10,), (10,10,), (50,), (50,50,), (100,), (100,100,)] |
| RF (no. of trees: 1000) | “max_depth” (max depth of the tree) | [2, 3, 4, 5] |
|  | “min_samples_split” (min no. of samples) | [2, 3, 4, 5] |

LR: logistic regression, SVM: support vector machine, SNN: standard neural network, RF: random forest
